# Understanding how older people with mild frailty engage with behaviour change to support their independence: a qualitative study

**DOI:** 10.1136/bmjopen-2024-086642

**Published:** 2025-01-30

**Authors:** Yolanda Barrado-Martín, Rachael Frost, Jessica Catchpole, Tasmin Alanna Rookes, Sarah Gibson, Christina Avgerinou, Benjamin Gardner, Rebecca L Gould, Paul Chadwick, Jane Hopkins, Vari M Drennan, Kalpa Kharicha, Louise Marston, Rashmi Kumar, Rekha Elaswarapu, Claire Jowett, Kate R Walters

**Affiliations:** 1 Centre for Ageing Population Studies, Research Department of Primary Care and Population Health, University College London, London, UK; 2 Bradford Teaching Hospitals NHS Foundation Trust, Bradford, UK; 3 Department of Psychology, University of Surrey, Guildford, UK; 4 Division of Psychiatry, University College London, London, UK; 5 Division of Psychology & Language Sciences, University College London, London, UK; 6 Patient and Public Involvement Contributor, London, UK; 7 Centre for Health and Social Care Research, Kingston University, London, UK; 8 Health and Social Care Workforce Research Unit, The Policy Institute, King's College London, London, UK; 9 Research Department of Primary Care and Population Health, University College London, London, UK; 10 Patient and Public Involvement Contributor, Birmingham, UK

**Keywords:** Frailty, Health Services for the Aged, Patient-Centered Care, PUBLIC HEALTH, Psychosocial Intervention, QUALITATIVE RESEARCH

## Abstract

**Abstract:**

**Objectives:**

To explore barriers and facilitators to behaviour change in older people with mild frailty.

**Design:**

Qualitative study.

**Setting:**

Community-dwelling older people living with mild frailty.

**Participants:**

64 older people with mild frailty, workers delivering the service and stakeholders.

**Methods:**

Semistructured interviews were conducted between July 2022 and May 2023 with participants in a randomised controlled trial (‘HomeHealth’) of a 6-month, home-based, personalised goal setting intervention, based around the Capability-Opportunity-Motivation-Behaviour model. We purposively sampled older participants receiving the service (n=49), workers delivering it (n=7) and stakeholders supporting its delivery (n=8). Interviews explored participation experiences, including engagement, perceived progress and impact. Transcripts were analysed using thematic analysis.

**Results:**

Key themes included frailty symptoms and adapting/compensating for these, self-efficacy and beliefs about capacity or need for change, familiarity with goal-setting processes and health-related knowledge, accessibility of services and outdoor environments, and enabling social support. Participants were empowered to change behaviours with support, where personalised meaningful goals were set. These were maintained where they led to a tangible outcome and had increased self-efficacy; however, new health challenges and lack of intrinsic motivation could be barriers.

**Conclusions:**

Regular and continued empathic person-centred support helps empower mildly frail people who are motivated to change their behaviour. Identifying those willing and able to identify their need for change may be key to maximise service use impact.

**Trial registration number:**

ISRCTN54268283.

**STRENGTHS AND LIMITATIONS OF THIS STUDY**

- Our qualitative study explores multiple complementary perspectives of interviewees, with a large sample of older participants, all support workers and stakeholders from voluntary sector organisation providers.
- The study was led by a separate researcher not involved in the development or the delivery of the trial and involved a wide range of people (including researchers and public contributors) with diverse backgrounds.
- The purposive sampling strategy over-represented the views of those who were less engaged with the service to ensure we captured negative views.
- During interviews, some participants struggled to recall details of the service and differentiate our service from other services received.

## Introduction

 Frailty is a condition characterised by reduced physical reserves and ability to recover from adverse events. Frailty is estimated to affect around 12% of those 50 years and over worldwide.^1^ Individuals living with frailty are at increased risk of falls, hospitalisations, death, decline in mobility and activities of daily living.^2^ Frailty is a continuum, whereby individuals can both improve and deteriorate, with potential for interventions that could reverse or delay frailty progression.^3^ Although services often target people with moderate and severe frailty, those at the early stages of frailty are more likely to become robust over time.^4^ Hence, there is a need to explore ways of halting the progression of frailty earlier on.

Different approaches have been used to reduce the impact of frailty, including exercise, nutrition, cognitive training and environmental adaptations,^5^ though there is insufficient evidence to recommend an optimally effective combination of these in the context of mild frailty.^6^ Strategies aimed at enhancing individuals’ motivation and capability to change their behaviour, including the use of goal setting or education, are a key element in the design of complex health promotion interventions, but have often been overlooked.^7^ To be implemented in practice with sufficient uptake, interventions should be acceptable to older people and aligned with their needs, perceptions and expectations.^8 9^ Research has shown that frail older people may resist frailty self-identification^10^ and tend to see frailty as non-modifiable,^11^ which may result in them not joining or benefiting from potentially relevant interventions. It is therefore important to understand how we can optimise engagement in interventions for early frailty, which is likely to differ to those living with more advanced frailty, as well as ways to promote behaviour maintenance longer term.

In a recent trial, older adults with frailty were unclear of the aim, potential benefits and behaviour change elements (goal setting and action planning) of a personalised care planning intervention for frail older adults.^12^ However, interventions for people living with frailty are generally highly acceptable and well attended.^13 14^ Nonetheless, detailed reports exploring what influences engagement in health promotion interventions for older people with frailty are rare.^14 15^ This limits our understanding of the factors helping or hindering engagement with interventions that could enhance independence in mild frailty.

We co-designed a health promotion service for older people with mild frailty (‘HomeHealth’), which aimed to promote independence and well-being. This was a home-based, tailored, six-session, multidomain (including mobility, nutrition, socialising and psychological well-being, among other domains) behaviour change intervention delivered by trained non-specialists employed by voluntary sector organisations. Details of the development of the service and content are reported in our earlier work^16^ and our protocol paper, including a Template for Intervention Description and Replication checklist.^17^ Results from a feasibility randomised controlled trial (RCT) were promising, suggesting that the service could increase independence.^16^ We further conducted a full scale RCT (the HomeHealth Trial) involving 388 people, 195 of them in the intervention group.^17^To facilitate behaviour change, the Capability-Opportunity-Motivation-Behaviour (COM-B) model of behaviour was used to find ways of promoting capability, opportunity and motivation.^18^ The clinical and cost-effectiveness results of the main trial has been reported elsewhere,^19^ as will the process evaluation qualitative data specific to the HomeHealth intervention itself (eg, acceptability, context, implementation) and quantitative data on reach, dose, fidelity, goal choice and progression.^20^

This paper reports on findings from a qualitative study, embedded within the HomeHealth trial exploring: (1) barriers and facilitators to behaviour change in older people with mild frailty and (2) how successful strategies to overcome these barriers were and what influenced this.

## Methods

### Design

Qualitative, semistructured interviews, embedded within the HomeHealth trial.^17^We followed the Consolidated criteria for Reporting Qualitative research Checklist (see online supplemental material A) reporting guidelines.^21^

### Patient and public involvement

We involved four public contributors with lived experience (JH, CJ, RK and RE). JH was involved in the study conceptualisation and funding acquisition, and JH and RE were involved throughout the pilot stage of this study. All four public contributors gave feedback throughout the trial and process evaluation and contributed to the formal analysis and writing of the manuscript.

### Participants and procedure

Data collection for this study was conducted between July 2022 and May 2023. To capture the views of all involved in the service, interviews were conducted with three types of participants: older adult trial participants who received the HomeHealth service, HomeHealth workers who delivered the service and service managers who supported delivery of the service.

#### HomeHealth service trial participants

Participants allocated to the intervention arm of the HomeHealth trial who consented to be approached for interview were eligible to participate. Trial eligibility criteria were: aged 65+years, community-dwelling, scoring as ‘mildly frail’ on the Rockwood Clinical Frailty Scale,^22^ life expectancy over 6 months and capacity to consent (see Frost *et al*^17^ for further details).

We purposively recruited participants for maximum diversity according to the following service characteristics: site (London, Hertfordshire, Yorkshire), HomeHealth worker, type of outcome goals set, face-to-face or remote delivery and service engagement (eg, number of sessions attended, setting or declining to set goals). We invited participants with diverse sociodemographic and individual characteristics, including gender, age, country of birth, ethnicity, sexual orientation, education level, baseline cognitive and physical functioning scores on Montreal Cognitive Assessment,^23^ Index of Multiple Deprivation scores and number of adverse events.

Potential participants were approached by post, with telephone follow-up by a study team member (JC, TR or SG) up to three times for non-responders. We completed interviewing older participants when data saturation had been reached, indicated by including participants with sufficiently diverse sociodemographic characteristics and a lack of novelty in interview content.

#### HomeHealth workers and other stakeholders

All HomeHealth workers who delivered the service were invited to interview, plus other stakeholders that were involved in service setup, hosting and supervision.

### Interviews

Each interview was guided by interview schedules tailored to each participant group (see table 1 and online supplemental material B), developed in consultation with the HomeHealth process evaluation team, and a Patient and Public Involvement (PPI) group supporting the HomeHealth trial (JH, CJ, RK and RE). Five researchers experienced in qualitative methods (YBM, JC, TR, RF, SG), with backgrounds in psychology, health services research and public health, conducted the interviews with older participants (see online supplemental material C). After the first seven interviews with trial participants, the interview schedule and procedure were refined in consultation with the process evaluation and PPI team.

**Table 1:** Interview schedules content by interviewee type

Older participants	HomeHealth workers	Stakeholders
Current health status, including potential long-term health conditions and memory difficulties, and perceived changes over the last year. Experiences of taking part in the HomeHealth study and participating during a pandemic. Experiences of maintaining behaviour change following their involvement in HomeHealth. Experiences around the HomeHealth service’s organisation. Perceived impact of HomeHealth. Recommendations for the HomeHealth service.	Motivations to work with the HomeHealth service. Previous work experiences and differences with HomeHealth. Experiences delivering the HomeHealth service. Adaptations while delivering the intervention. Experiences of working during the COVID pandemic. Perceived impact of the intervention on participants. Fit and integration within VSO structures. Experiences of training and supervision received. Recommendations for future implementation.	Understanding of HomeHealth, differences with other services, and motivations to incorporate this service within their Voluntary Sector Organization (VSO). HomeHealth’s fit with their VSO. Experiences setting up the service. Training needs, access to topic experts’ advice, and supervision delivery experiences. Recommendations for wider implementation of the service.

Initially, interviews with trial participants were completed after 12-month follow up (6 months post-intervention; see Frost *et al*.^17^). As some participants found it difficult to remember details of the service, we were granted an ethics amendment to interview participants post-intervention but prior to their 12-month follow-up assessment.

Interviews lasted 68 min on average (range 35–124 min) for trial participants, 118 min for HomeHealth workers (range 96–136 min) and 60 min for stakeholders (range 48–81 min). All interviews were audio-recorded, professionally transcribed verbatim and pseudonymised. Interviews were not returned to participants for comment.

### Analysis

Interview transcripts were analysed using codebook thematic analysis^24^ in NVivo 12.^25^ Two researchers (YBM, TR) coded three interviews and developed a preliminary coding framework inductively that was presented and discussed with the study team and finalised following discussions among five researchers (YBM, RF, TR, JC, KW). Interviews with stakeholders and HomeHealth workers were coded by YBM, and interviews with older participants were coded by JC, TR, SG, YBM and RF. All interviews were coded using the same framework, with differences in each type of participant’s contribution to the different codes. Coding was regularly discussed in team meetings to ensure consistency and to adapt the framework where needed. Subthemes were developed by YBM, presented to the study team and further refined through discussions among study team members including discussions with PPI members. These were then grouped using the COM-B model which underpinned our intervention, to outline the main factors affecting behaviour change and ways these could be overcome. Themes specific to the mixed-methods process evaluation on intervention acceptability and contextual factors affecting implementation are summarised as part of a separate paper alongside quantitative process data.

### Ethical considerations

The HomeHealth trial (including this embedded qualitative study) was approved by the Health Research Authority Social Care Research Ethics Committee (ref 20/IEC08/0013). All participants provided informed consent to participate, either written (for face-to-face interviews) or audio-recorded oral consent (for remote interviews).

## Results

### Participant characteristics

Of 84 participants approached for an interview, 14 declined to participate (with reasons including not wanting to take part in interviews, not having anything to add or not being the right time), and 16 did not respond. Fifty-four expressed an interest, of whom six were not interviewed for different reasons (eg, died, illness/hospitalisation, contact was lost, public transport strikes). One additional participant contacted the research team without an invitation, expressing interest in taking part in an interview, and was also interviewed. Thus, 49 interviews were conducted with HomeHealth participants.

Interviews with HomeHealth participants (n=49) were mostly conducted face-to-face (n=43) at participants’ home, with four conducted over the phone and two via video-conferencing. Most trial participant interviews were conducted on a one-to-one basis (n=46), with three dyadic interviews with participants who chose to have their carer present. In total, 29 interviews were completed after the 12-month follow-up window period (−2 to +4 weeks of the date 12 months from randomisation), six interviews during the 12-month follow-up window period and 14 interviews before the 12-month follow-up window. Older adult interviewees had a mean age of 80 years, were more commonly female (65.3%), born in the UK (69.4%), of white ethnic background (83.7%), heterosexual (93.9%), with no formal qualifications (38.8%) and with diverse levels of deprivation (table 2). Cognition scores typically suggested mild cognitive impairment, functional scores suggested slight dependency, and most decided to pursue mobility goals or a combination of goals most commonly including mobility.

**Table 2:** Characteristics of older participants interviewed

Characteristics	Frequency/mean (SD, min, max)
Number of appointments	5.5 (SD=0.84, min 3, max 6)
Age (at recruitment)	80.3 (SD=6.58, min 66, max 94)
Gender	
Female	32
Male	17
Born in…	
UK	34
In another country	15
Ethnicity	
White: English/Welsh/Scottish/Northern Irish/British	37
Any other White background	4
Black/African/Caribbean/Black British: African	2
Asian/Asian British: Indian	2
Irish	1
Any other Asian background	1
Any other mixed/multiple ethic background	1
Any other ethnic group	1
Sexuality	
Heterosexual	46
Homosexual	1
Prefer not to say	2
Highest level of education	
No formal qualifications	19
General certificate of education/O levels or equivalent	11
Certificate of secondary education/A levels or equivalent	2
Higher national diploma or equivalent	3
Degree	8
Higher degree	6
Index of multiple deprivation (possible range 1–10)*	5.3 (SD=2.91, min 1, max 10)
Baseline Montreal Cognitive Assessment (possible range 0–30)†	24.1 (SD=3.63, min 16, max 30)
Adverse events	1.7 (SD=1.93, min 0, max 10)
Falls	1.2 (SD=1.7, min 0, max 10)
Baseline Barthel (possible range 0–100)‡	95.7 (SD=6.17, min 63, max 100)
Goal types	
Mobility only (exercise/stay independent and active/walking)	20
Mental health and well-being only (managing mental health/finding motivation/finding or carrying out meaningful activities)	4
Social only (maintaining social life)	2
Other	2
Combinations of different goal types, mostly including mobility	17
No goals or no data	4
Goal progress	1.21 (SD=0.55, min 0, max 2)

* Higher scores indicate less deprivation.

† Higher scores indicate better cognitive functioning.

‡ Higher scores indicate higher independence levels.

All HomeHealth workers who delivered the service (n=7) consented to take part in an interview following an email invitation. Ten stakeholders were approached via email, eight of which responded and were interviewed. The eight interviewees included six people involved in service setup and supervision, the HomeHealth team leader and an expert who provided exercise training and supervision. All HomeHealth workers and stakeholders were interviewed online via video-conferencing.

HomeHealth workers’ and stakeholders’ detailed demographic data were not collected. All HomeHealth workers were female, five from White backgrounds, one Black and one Asian. They had professional backgrounds in health and social care, but one person had a background in finance. All were trained in delivering the service. Among stakeholders, there was only one male, one was from Asian background and the rest were from White backgrounds.

### Themes

Participants identified a range of barriers and facilitators to behaviour change and approaches to overcome challenges, which we have grouped under elements of the COM-B model.

### Capability

#### Frailty and health problems

Physical capability had a clear impact on participants’ goal achievement, with many participants experiencing health problems as part of mild frailty. Fatigue and lack of energy, or symptoms such as pain, affected participants ability to carry out goals which required regular behaviours or to progress goal intensity, particularly those relating to exercise or more active daily activities such as shopping. Participants also highlighted fluctuations in health conditions or acute illness that led to temporarily ceasing activities. These health issues were attributed to old age or long term conditions, and so participants felt little could be done to mitigate these.

*I had a good day when we went to the pantomime and then the next day was okay. Then whatever I’ve got now, I don’t feel good, I don’t feel ill. It’s something, you’ve got to live with it and I think, Well okay, so does everybody else but you can’t progress much, I don’t feel but there you are*. (TP02134, F, 75-84yrs)

However, this was not a major barrier where participants were sufficiently motivated. Many had developed strategies to manage the effects of these in order to continue their everyday life, and so building on these strategies was a helpful way to overcome these barriers. This could be through carrying out a new behaviour at an optimal time (eg, when having more energy), using assistive equipment, temporarily reducing activity or exercise levels when they lacked energy or were in pain, pacing activities or adjusting the goal to a lesser amount.

*If my shoulders hurt, I’ll do it [the behaviour] tomorrow or whatever. I don’t beat myself up if I haven’t done them.* (TP03101, F, 65-74yrs)

*Because of the limbs aching sometimes even the exercises are limited to not repeating many times, only once or twice rather than five or 10 times*. (TP02021, M, 85-94yrs)

HomeHealth workers tried to overcome these challenges by signposting to healthcare professionals (eg, a general practitioner (GP)) for medical issues (eg, pain) and recommending assistive equipment such as mobility scooters. They found it important, but sometimes challenging, to distinguish between pain caused by a health condition, and pain caused by exercises.

Some participants dropped the goal entirely as they were physically unable to perform the activity or had tried it and experienced negative consequences (eg, joint pain).

*[Standing] on one leg, was another one that we did. I don’t do that [exercise] anymore, I keep falling over*. (TP03038, M, 75-84yrs)

This was unproblematic if participants felt positive that they had tried; it was one of a set of goals or was seen as less important. However, in a small number of cases, participants found it upsetting to realise they were physically unable to achieve a goal.

*Because of the knee, I can’t swim properly, I can’t. That upset me. I was upset about that because I really thought the water would be very good, and swimming, and meeting other people, but it didn’t work*. (TP01046, F, 75-84yrs)

#### Self-esteem and self-efficacy

Self-esteem and self-efficacy were particularly strong barriers for some:

*I knew something needed doing but I didn’t know how to start it and my brain wouldn’t tick over enough.* (TP03101, F, 65-74yrs)

Where people had sufficient self-efficacy to make changes, whether this occurred depended highly on motivation levels. For those who wanted to change but had low self-efficacy, an individualised approach with a dedicated person to listen to them allowed them to focus on their own needs and feel they mattered, sometimes over the ones of others around them (eg, spouse). Some also reported feeling it gave them space to think through and decide on potential solutions. This led to feeling an increase in their self-worth and sometimes finding a sense of purpose they thought they had lost, which facilitated behaviour change.

…*She [HomeHealth Worker] encouraged me, and she told me that I am [good at art]. It’s surprising, instead of having that critique, having somebody to boost your morale and this is what she did.* (TP03036, F, 65-74yrs)

Some participants did not see the need for health promotion for themselves; they felt they were ‘good enough’ and in some instances more fortunate than others around them in terms of their ability to make changes without help and support available, though viewed the service as something to benefit from if they were worse off in the future. They felt services to support independence would be more beneficial to others with more complex needs, less social support and self-efficacy

*someone who’s not so strong-willed as I am [and] perhaps need[s] more help* (TP02111, M, 75–84 years).

#### Familiarity with goal setting

Familiarity with the language of goals and the goal setting process was also a factor. Those who were familiar or open to goals engaged well with the process and terminology and found it helpful. Others found the language and processes off-putting but were happy to remain engaged with the service. In these cases, HomeHealth workers reported changing the language (eg, to actions or targets), trying to get participants to understand how they were already using these techniques in everyday life, focusing on the conversation rather than writing down goals and breaking down the goals.

*There were a few participants where, actually, they were like, point blank, they don’t want to set goals. So, we’d have to set, kind of, actions, or targets. But it would be very much in the conversation. Like, they didn’t want anything written down*. (HomeHealth worker 2)

They reported this worked successfully in some cases. Where it did not, HomeHealth workers typically moved towards a case management approach, where they more actively supported participants to take actions, for example, link with support services.

#### Knowledge

While lack of knowledge was only explicitly stated as a barrier by a few participants, a substantial number reported that the support worker had signposted them to useful services that they had not previously been aware, and most reported positive experiences from these.

*The information that she gave me, that she got, was so useful*. (TP01046, F, 75-84yrs)

Feeling knowledgeable could also be a barrier, in that those who felt very knowledgeable about independence and local services they felt the HomeHealth worker had little to add unless practical recommendations were made.

#### Beliefs about capacity for change

Some felt that ageing was a downhill process and little could be done to prevent decline. This influenced their self-efficacy and motivation to make changes. While the HomeHealth worker could provide social support and empathic listening, initiating behaviour change and overcoming capability barriers were challenging.

*There’s nothing she could do about what the situation is. She can’t make you any younger, she can’t make you healthier, as much as she would be willing to do sort of thing.* (TP02132, F, 85-94yrs)

However, for other participants, a preventative approach aligned well with their perceptions of ageing and fears of future loss of independence, and perhaps consequently, they expressed positivity about preventative services and engaged well with HomeHealth.

*…[HomeHealth is] preventative…and it’s perfect for me really because I don’t want to go in a home. I would hate that.* (TP02118, F, 75-84yrs)

### Opportunity

#### Access to services

The accessibility of services and activities affected opportunities for behaviour change. Transport was a particularly strong barrier for those with restricted mobility.

*The day centre is probably about a mile and a half away […] I can’t bloody walk it*. (TP02134, F, 75-84yrs)

The main way of overcoming these barriers was enablement, through taking an active role in connecting people with local services (eg, arranging to start a class, booking a GP appointment) or providing information on transport services and benefits. Support workers also built participants’ confidence to overcome access barriers, such as empowering them to regain confidence to insist, so they could be seen by professionals in person where needed.

…*she’s saying, “Ring him [GP] up and find out [if they can help you]. Get on with it,” and so I did at the finish. I found that, like I say, she was encouraging me to be proactive, do something, don’t just sit and accept it, so I did.* (TP03098, F, 65-74yrs)

Access to services and activities was strongly affected by COVID-related service restrictions during the study. Although some services were re-opening later during the time the service was run, some participants reported being unable to (re)join services due to ongoing or permanent closure after the pandemic. Cost was rarely mentioned as a barrier, apart from in relation to services to aid mobility such as taxis. This perhaps reflects the fact that most goals set were not ones which required people to pay or to use resources.

#### Accessibility of the local outdoor environment

Time of year (ie, hours of daylight available) and associated adverse weather conditions were further barriers to behaviours such as walking more or attending activities, as participants were put off by the slippery surfaces, lack of security or the negative impact of extreme weather on symptoms. In response to this, activities were mostly ceased.

*I’m not going to do the art group. I’m not even attempting it until the weather improves*. (TP02134, F, 75-84yrs)

However, some participants moved to indoor alternatives for exercise, such as walking around the house or using an exercise bike. Drier and sunnier weather and getting involved in group activities were seen as incentives for behaviour change.

#### Time and competing commitments

Time availability and the impact of competing commitments were also relevant. Some juggled caring responsibilities or complicated (family) life events at the time the service was being delivered. For these kinds of barriers, support workers reported postponing behaviour change or focussing on supporting with the life events instead where possible.

…*My son in law died and since then until now I’ve been more than tied up with helping my daughter cope with the, attempting to cope with the grief so that has changed everything. I haven’t been able to focus enough on me*. (TP01003, F, 75-84yrs)

#### Social support

Beyond physical accessibility, having access to an empowering, pressure-free network of supporting others (ie, family, friends, HomeHealth worker) was also critical for behaviour change and overcoming physical barriers such as lack of transport. Some participants and HomeHealth workers reported good family support helped with prompting or reminding participants to do a behaviour, but for others, their support network could be lacking or discouraging (eg, questioning their ability to do things outside their usual environment). For a few women, their husband’s health or reluctance to do things were particular barriers:

*I put our name down for it [to go on a trip] and my husband, when we came back here, said, “(…) What if you can’t manage the room? What if we can’t get a downstairs room? What if-“…(…) So I said, “Forget it. I’d sooner not bother with it than go”.* (TP02132, F, 85-94yrs)

The social support from HomeHealth workers provided the opportunity to consider making changes and how potential barriers might be overcome, emphasising the importance of the changes through monitoring goal progress and giving people the opportunity to discuss all relevant aspects of their life:

*Almost every single person I saw was down, and really having somebody to talk to was a goal but they don’t tell you that (…) They want to have a human being to talk to, so I think again it’s like there’s a sort of goals that we’re allowed to talk about, that are sort of publicly acceptable.* (HomeHealth worker 4)

Participants were reluctant to give up this social opportunity that allowed them space to explore and set goals and as a point of contact for further help.

*I’ve had people say to me, “Oh is it only six visits? Can you not like carry on? (…) We don’t expect you to come every week but even if it’s once a month you know it’s comforting and reassuring that you know there’s somebody there if you need help”.* (HomeHealth worker 6)

### Motivation

#### Perceived need for change

Where participants had clear expectations, needs or something they wanted to work on from the outset, they were motivated and enthusiastic about making changes and persevered with goals despite setbacks such as weather and ill-health. The HomeHealth workers supported them to reduce the number of goals or break down their larger goals into realistic ones.

*What I wanted to achieve was swimming (…), reading, (…) [and] to keep friendships going.* (TP01046, F, 75-84yrs)

As well as positively impacting capability, being listened to by HomeHealth workers and feeling they mattered also motivated participants to make changes.

Others were fairly active with hobbies and socialising and content with their present life and did not feel they wanted or needed change and had joined the project to contribute to research rather than from perceiving a personal need.

*I haven’t got any priorities. (…) She couldn’t offer me what I wanted because I don’t know what I want.* (TP02111, M, 75-84yrs)

#### Identifying goals

Many participants initially struggled to identify goals and took time to identify them. Support workers reported trying to overcome this by spending the first appointment getting to know the person and focussing on goals at the following appointment. For some, they preferred a day-to-day focus rather than setting medium-term goals, as they felt the future was uncertain and that health problems were only likely to worsen.

Where participants felt content with their current life with little motivation to change, HomeHealth workers focused on encouraging them to maintain current activities and supporting them to identify ways these might be made easier (eg, by improving grip). Participants generally viewed this as helpful but could report consequently that they felt they had gained little from the service. Occasionally, when the participant persistently struggled to identify a goal, HomeHealth workers took a more leading role, suggesting goals that might fit the individual’s circumstances. This had variable success**—**people reported being willing to try something new but did not always find it helpful.

*With HHW1B I agreed to go to the coffee afternoon, I did but I wouldn’t say it really helped.* (TP03086, 2, 65-74yrs)

The goals set needed to align with people’s innate or longstanding preferences, which were explored through comprehensive discussions with support workers.

*I just don’t really want to start going out anymore to these groups, where there are groups of people. I’ve never been very much of an extrovert type of person*. (TP03055, F, 65-74yrs)

### Changing behaviours

#### Impact and reinforcement

Over the course of the intervention, there were clear impacts from the achievement (or not) of behaviour change goals. Most interviewed participants progressed in at least some of their goals. The positive progress made by some participants increased their self-efficacy for further changes, their motivation to set and pursue new goals, and their motivation to continue behaviours with a tangible positive outcome. It also gave them the opportunity to refine their priorities.

…*When people do successfully achieve the goal, you can really see the impact on their life, that they’re more willing to try things, and try things that-like, thoughts that would have in their mind that they never got around to doing*. (HomeHealth worker 2)

*And it’s [HomeHealth] improved my life (…) The team have given me so much confidence, help and I don’t know where I’d be without it because I think I’d have just gone further back.* (TP03101, F, 65-74yrs)

Generally, participants who chose to do a tailored set of strength and balance exercises found them helpful in promoting their independence and improving their functioning, and this led to maintenance of the behaviour, at least to some extent.

*[I do the HomeHealth exercises] Not as often as I should to be perfectly honest, but I think they’d be more helpful, really quite helpful if I did them more often*. (TP02134, F, 75-84yrs)

A few participants had not been able to achieve all their goals set during the service and reported they were still working towards them at the point of the interview. Some participants felt that they ‘should’ have carried out the behaviour more frequently and expressed a level of guilt and self-blame despite often attributing this to clear capability or opportunity barriers. HomeHealth workers generally addressed this as a problem of intrinsic motivation and highlighted the need to support participants to move away from the expectations that they should meet certain standards to make behaviour change possible and sustained.

…*Moving away from “shoulds” as well, so trying to encourage people to be doing behaviours- and it could be because they think it’s useful for them in the long-term but doing it because they’re wanting to rather than because-again it’s, like, internal locus of control versus somebody outside telling you. Like, feeling the benefits of that in whatever way that is*. (HomeHealth worker 4)

For participants who had not been able to achieve their goal, HomeHealth workers tried to build motivation through assessing why they wanted to carry out the goal and addressing any further capability or opportunity barriers. Where behaviours had shown a negative impact (eg, increased pain), participants reported ceasing the goal during the intervention or adapting it (eg, reducing recently increased exercise intensity). Where behaviours showed no or little impact, participants sometimes continued while seeing a HomeHealth worker, but rarely reported maintaining these after the service.

*She got me an egg to squeeze to try and improve my hand. She brought that up when she came. The biggest problem is that it’s a nerve that’s causing the problem so it didn’t really make a huge difference*. (TP03098, F, 65-74yrs)

*But [the HomeHealth worker] it was just simply incentive, not say exactly pleasing somebody but along those lines.* (TP01044, M, 85-94yrs)

#### Maintaining behaviour longer term

The main maintenance challenges were often reported to be capability-related, with the onset or worsening of new symptoms or health conditions, which could be difficult for participants to address without further external support. Intrinsic motivation, including having the energy, drive, and determination to do the exercises, and enjoyment of a behaviour, helped to maintain the behaviour beyond the service. Incorporating behaviour changes in their normal daily routines also helped (eg, using exercise equipment while watching TV).

*[I do the hand strengthening exercises] Probably most evenings as I’m sitting there (…) and they seem to loosen up my joints.* (TP01112, F, 85-94yrs)

HomeHealth workers reported encouraging maintenance after the end of the service through forward planning and supporting self-efficacy.

…*When obviously the sixth session is coming to an end I said that you know obviously they continue with these exercises and if they feel they’ve reached capacity like they can’t do anymore repeats you know just to continue with those repeats and (…) increase the repeats if they can*. (HomeHealth worker 6)

## Discussion and implications

Older people with mild frailty engaged with a home-based behaviour change health promotion service, but the degree and type of engagement varied according to a range of factors. Physical and psychological capability to change were important influencing factors, including health and frailty symptoms, for example, fatigue or pain, and psychological factors including self-esteem and self-efficacy. Barriers in opportunity largely related to accessibility of services and outdoor environments in the context of impaired mobility of participants. Service availability was also impacted by the after-effects of the COVID-19 pandemic on services at the time of the study. An important facilitator was the involvement of supportive others (family, friends and the support worker themselves) in behaviour change actions and facilitating access to other services. Similar barriers in health symptoms limiting capability to be physically active and in access to services have been found in qualitative studies exploring older adult’s participation in physical activity more generally.^26^ Motivation to change was clearly influential, with facilitating factors including a self-identified need for and perceived ability to change, alongside the perceived relevance of setting goals and the importance of the goals to their lives. The person-centred and asset-based approach were deemed particularly helpful and empowering. Regular contact with and continuity of support workers over six sessions was seen to help with developing rapport, person-centred goals, enabling participants to over-come some of these barriers, and filling a gap in services.

In this study of older people with mild frailty, some felt a health promotion service would be more useful for others with less social support or greater frailty. Other qualitative studies also suggest that older people with mild frailty often compare themselves positively to others who are worse off,^27^ attributing the label of frailty to others who are more unwell or who have lost their independence.^28^ We found that some individuals found the process of goal setting challenging. This has also been found in other work, for example, in feasibility studies using Goal Attainment Scaling in practice with older people in the Netherlands, some struggled to identify their goals,^29^ and in the USA, some older adults did not feel comfortable talking about potential negative future outcomes.^30^

An important part of any behaviour change intervention is the promotion of maintenance of the new behaviour over time.^31^ Participants often reported they had continued practising their new behaviour, facilitated by incorporating it into their daily routines and using prompts to remind themselves. Participants who autonomously decided on goals that were relevant to them, felt able and competent doing a behaviour that was aligned with their values and interests (intrinsically motivated), appeared more often able to maintain the behaviour over time. This aligns well with self-determination theory.^32^ However, those that relied on external motivators to do the behaviour (such as the support worker) or picked the goal according to what they felt ‘should’ be doing (extrinsically motivated) struggled to maintain the behaviour. Further support and behavioural planning are likely to be needed for this subgroup, since it is unlikely that all frail older people are intrinsically motivated to proactively optimise their health.^26^

### Strengths and limitations

Strengths of this study include the multiple complementary perspectives of interviewees, with a large sample of older participants who varied according to intervention engagement and sociodemographic characteristics, all support workers and stakeholders from Voluntary Sector Organization providers. The study was led by YBM, a separate researcher not involved in the development or the delivery of the HomeHealth trial, and involved a wide range of people (including researchers and public contributors) with diverse backgrounds.

However, the purposive sampling strategy over-represented the views of those who were less engaged with the HomeHealth service to ensure we captured negative views and, hence, findings need to be considered carefully. Additionally, during interviews some participants struggled to recall details of the service and differentiate HomeHealth from other services received. This has been reported in another study^13^ and was probably exacerbated by the high prevalence of participants living with mild or moderate cognitive impairment according to the Montreal Cognitive Assessment scores^23^ in our sample.

### Implications

Targeting behaviour change through strategies to promote motivation and capability to change, such as person-centred goal setting, appears to be possible in a mildly frail population. However, in some cases, it can be challenging, with barriers including conceptual understanding of the aims and processes, intrinsic motivation to make changes and physical capability to commit to behaviour change and make the desired progress. In those where it worked well, motivations included determination to remain independent at home and reduce future potential burden on families and self-identification of a behaviour change need and access to material (eg, services) and immaterial (eg, social support) resources. For those that struggled with goal setting, a more case-management and empathic listening approach was perceived as being of value. Where goal-setting with action planning is being incorporated into routine care, for example, for those with multiple long-term conditions, or where addressing person-centred care for the National Health Service long-term plan,^33^ older adults may need support with understanding the purpose and identifying a need for change in the first instance. Once this is established, sufficient time is needed for a person to fully understand their goals and needs, and maintenance of any changes needs careful planning, particularly in those with external locus of control. Holistic person-centred services allow initial barriers (such as linking in with existing support structures) to be overcome more easily.

Future work should explore individualised versus group approaches in older adults with mild frailty. Understanding, recognising and perceiving frailty as amenable to change are required for older people to feel they can act on it. Further work is needed to understand how best to raise awareness and address this in practice.

## supplementary material

10.1136/bmjopen-2024-086642 online supplemental material 1

10.1136/bmjopen-2024-086642 online supplemental material 2

10.1136/bmjopen-2024-086642 online supplemental material 3

## Data Availability

Data are available upon reasonable request and subject to ethical requirements on data sharing.
