# Supplementary material for: Understanding how older people with mild frailty engage with behaviour change to support their independence: a qualitative study
Source: BMJ Open. 2025 Jan 30;15(1):e086642. doi: 10.1136/bmjopen-2024-086642 (PMC11784424; doi:10.1136/bmjopen-2024-086642)
Supplement: online supplemental material 1 [file bmjopen-15-1-s001.docx]

# Supplementary material B – Interview schedules per participant type

**Topic guide for interviews with older people**

*The HomeHealth service is a new service. We are talking to people who received the HomeHealth service to find out what they thought of it, which parts of the service worked better, and what did not work so well, so we can improve it. We are very keen to hear your views, so your honest feedback is really important to us. And just to note, nothing that you say here will get fed back directly to the people you saw during the trial.*

- *Have you read the information sheet? Was the information clear?*
- *Do you have any questions?*

| Instructions for interviewer |
| --- |
| Confirm confidentiality - data will be anonymised |
| Check consent form is completed |
| **Switch the audio recorder on** |

1. **Opening: Warm up -** Let’s start out by letting me find out a bit more about you.

- Can you tell me **how your health is at the moment? / How has your health been recently?**
  - Do you have **any long-term health conditions**?
    - Can you tell me **what they are**? It’ll help later on.
    - How do they **affect** you/your **everyday life**? (prompts: medications, health appointments, symptoms, mood-wise)
- How has your **typical day changed** since you started the study in [month and year]? (i.e., daily activities, health, memory).
- How common is it for you to forget things, have difficulty concentrating or finding your way around?
  - How do these difficulties affect you and your everyday life? (prompts: need for support from others, difficulty with admin tasks, how self-manage, probe on specific issues e.g., finding way around)
  - How has your memory changed, if anything, in the last year? [probe on specific changes, explanations for these, impact of Covid, has anyone around you noticed/mentioned changes]

1. **Taking part in the HomeHealth study - [Prompt: Flowchart]** As part of the HomeHealth study, you had some research assessments and then you saw <HHW> for about six months, you talked with her about priorities and ways to maintain your [mobility/wellbeing/mental health etc]. Does that sound about right?

- Do you **recall identifying** any problems that you wanted to work on with <HHW> to maintain your [mobility/wellbeing/mental health etc]?
  - [If no, they didn’t set any goals]- **Why** was that?
    - What kind of things did you **discuss** with the HomeHealth worker in the appointments?
    - What **could have helped** you finding something you wanted to work on or achieve?
  - [If no, they don’t remember] – **I have some information** here that the HH worker has shared with me. It suggests that you worked on [state whatever goal is mentioned]. Do you remember talking about this with the HH worker?
    - [If no] – No problem. Common things people may have chosen to discuss were their [mobility/wellbeing/mental health etc]. Is this something that you would have liked to work on. (probe: if yes or if no, why?)
    - [If yes – Continue with questions below].
  - [If yes, they do remember]- If so, what were these?
    - How did you **decide** on these priorities? (prompts: strength, pandemic, fatigue)
  - How easy was it to **find something** you wanted **to work on?**
    - What helped you to **work towards** your priorities/aims?
  - Did your priorities **change over time**? In what way?
- Overall, **how much progress** do you think you made with your priorities?
  - - Probe: satisfaction with this, **impact** on you/your health and well-being
- What kind of things **got in the way** of you working towards these priorities? (e.g., boredom, lack of energy, not enough time, health problems, memory, hospital appointments, too much effort)
  - - Was there anything that could have helped you overcome these?
    - Was there anything HHW could have done?
  - What else **would you have liked** to work on?

We talked earlier about **long-term health conditions**. Sometimes people find managing a number of different long-term conditions challenging.

- Did you **talk** to the HomeHealth Worker **about these**? What sort of things did you discuss?
  - [If they set priorities] Did they **influence what you chose to work on**? If so, in what way? (e.g., importance of priorities, modifying priorities, unable to do certain priorities i.e., exercise)
- [If they did not set any priorities] How did they **influence your decision** to not work on any specific area to maintain your [mobility/wellbeing/mental health etc])?
- HomeHealth aims to help people maintain their independence rather than focussing on a specific health condition. What did you think about this kind of **approach**? (prompts: holistic, single point of contact, was health felt to be in HHW remit?)

We talked earlier about your **memory difficulties**.

- Did you **talk** to the HomeHealth Worker **about these**? What sort of things did you discuss?
- How did you find **working on your memory difficulties**? What **impact** did they have for you?
  - - - [If helpful] in what way?
      - [If unhelpful] why? anything could have been done that would be more helpful or would help with this?
- Did these problems with memory make it **more difficult to** **choose areas or priorities** to work on in the HomeHealth Service? In what way? (Prompts: deciding what to do, making plans, paperwork).
- How did you deal with this? Was there anything that made it harder for you? Anything that made it easier?
- What did you do to try to ensure you **remembered** to do [priority]?
- What additional support could have been provided to help you make progress towards… “your goal”? [Prompts: calls in between sessions, more sessions, others attending sessions, a session focussed on memory, reminders and alarms, equipment provided e.g. pillboxes]
- Did your HomeHealth worker **refer** you to/ **give you information on** any other services that could help you? (e.g., counselling, financial support/aids etc). How did you find this?
- Were there any **other changes you made** as a result of working with [Homehealth worker]? [prompts: during, after the service]

1. **Maintaining changes**

- Over the last [N] months or so since you last saw [HomeHealth Worker], have you **continued working** on [priority]? Or set any **new priorities/things you want to achieve**?
  - Can you tell me more about this?
  - Prompts: health conditions, memory, support from others
- Did you go on to explore any **local groups** (i.e., join an exercise class or walking group, interest groups)?
- Did you see **anyone else to help** you with your priorities or things you want to achieve after the HomeHealth worker sessions ended (e.g., social prescribing link worker or someone else)?
- What about informal **support from friends or family**? How did they help?
  - - How helpful was this?
- What **additional support**, if any, might have helped you to continue to work towards these priorities/ things you want to achieve? (e.g., ‘booster’ sessions with HH worker/telephone call, alerts/reminders, involving a family member, peer support).
- What might help you maintain your priorities (goals/aims) in the future?
  - What additional support may you need in the future if your health/memory get worse?
  - If another person had similar health/memory issues to yours, but was struggling more, what things do you think would help them?
- What would be the best way for people to access these?

1. **Experiences of the HomeHealth service organisation** - Again, I won’t tell <<HH worker>> what you say here, so you can speak freely.

- What do you think about how the service was **organised** (e.g., convenience, time, length, frequency, location, flexibility to re-schedule)? How were the **appointments** arranged?
  - How did you find the **contact** you had with the HomeHealth Worker **between appointments**?
    - - Would you have liked more or less contact between appointments? If so, how and why?
  - You had [number of] **sessions** with the HomeHealth Worker. Was that the right amount? (more/less)
    - - Did you manage to attend as many sessions as you initially planned? Why/why not?
    - The **role of the HomeHealth Worker** was developed specifically for this service. What did you think about your HomeHealth Worker? (i.e., communication style, skills, knowledge).
      - How did you **get on** with your particular HomeHealth Worker (rapport, empathy, style)?
      - Would you have **preferred a choice** (in terms of older age, gender, ethnicity faith etc)?
      - [If there was a change in HomeHealth Worker] How did you find having appointments with two **different workers**?
- Did **anyone else** attend the appointments with you e.g., partner, family member or friend?
  - How did they **support** you during appointments?
  - Did you find this **helpful**/would you have found this helpful? In what way?
  - Were there any times when having your partner/family member/friend there was **less helpful**? In what way? Was their presence **off-putting**? (Intrusion into ‘me’ time)
- What did you think of the health and wellbeing plan you completed with the HomeHealth worker?
  - How **helpful** did you find it? How easy to **understand** was it?
- [If applicable] At the end of your time with the HomeHealth service you may have been sent a **summary letter** of what had happened, that was copied to your GP. Due to some organisational issues this did not happen for everyone. Did you receive one? How did you find this letter? Was there anything that could have improved it/you would change?

1. **Experiences of HomeHealth during the Covid-19 pandemic** – Due to the Covid-19 pandemic, the service was only available remotely (i.e., via video or telephone call) in early 2021. When it was available face to face, HomeHealth workers tested regularly and used PPE.

- Were your appointments **remote or in person or both**?
  - How did this option work for you? Would you choose it again? Why/why not?
  - [If in person] How was it **having somebody visiting** you at home (during the pandemic)?
  - [If remote] Were they via **video or telephone** call?
  - [If remote] How did you find **remote appointments**? (prompts: technology, setup, ease of talking to someone by video/phone, support needed)
- During **Covid-19** pandemic people made a lot of lifestyle changes (prompt eating and drinking, socialising, shopping, worry?)
  - Did the COVID restrictions make you have to change how you worked on the priorities you set during your time in the HH trial? How so?

1. **Impact & recommendations for the HomeHealth service**

- How would you rate your **overall experience** taking part in HomeHealth?
- Why did you decide to **take part** in the study?
  - Should we write to people from their GP in future? If not, how should we let people know about it?
  - If it was just offered by Age UK or another voluntary service, would you still use it?
  - If you had to pay for the service—whether that was through the NHS or another service, would you still do it? If so, how much would you be willing to pay?
- Thinking back, before meeting [HHW], what were you **expecting** from HomeHealth?
  - Did you **get all** that you **had hoped** to get out of the service?
    - [If no] - Why do you think that is?
    - How did your health affect what you got out of the service?
  - Was there anything that **didn't meet your expectations**?
    - [If yes] How could these have been met?
  - [If, “I did not have any expectations/I didn’t know what to expect”]:
    - What additional information could have been provided to help guide your expectations and understanding of what to expect from the service (prompts: more written information at the start, having an end goal of success, discussing what is on offer and your needs/expectations with a member of the team)
- What did you find most **helpful** about the service?
- What did you find **unhelpful or** **difficult** about the service?
- How could we **encourage others** to use the HomeHealth service?
- Who do you think **needs** the homehealth service?
  - How could we let them know about it and encourage them to use it?
- How would you **describe** the **HomeHealth** service to a friend/family member?
- Would you **recommend** this service to a friend or family member who was experiencing similar difficulties to you? Why/why not?

**Summarise interview.**

- Is there anything about the HomeHealth service we could improve, that we haven’t already talked about?
- Is there anything else you would like to say?

**Thank you for your time and help.**

| Instructions for interviewer |
| --- |
| Switch audio recorder off |
| Give voucher |

**Topic guide for interviews with HomeHealth workers**

*Thank you for agreeing to take part in an interview. We are interested in your experiences of delivering the HomeHealth service, both good and bad, so we can improve the service for the future if it is implemented. We will keep your responses as confidential as possible - we will not feedback individual responses to either the team leader or your voluntary sector supervisors but will provide an overall summary of HomeHealth worker feedback. You will not be identified by name or organisation/area in any publications, but as there are only a small number of you, we cannot guarantee complete anonymity. You can stop the interview at any time. We would like to audio record the interview. Do you have any questions before we start?*

| Instructions for interviewer |
| --- |
| Check consent form is completed |
| **Switch the audio recorder on** |

*Note: [VSO] should be replaced with Age UK Camden, Age UK Bradford, HILS as appropriate*

**General**

- What first **attracted** you to working on HomeHealth? (i.e., working with older people, flexible hours, local, gaining experience and training in the field)
- Talk me through your **understanding** of the HomeHealth service (e.g., aims, content, delivery).
- How does HomeHealth **differ** from services you have worked in before?
  - How did the **nature of the relationship** (equal partnership between you and the older person) differ from the usual jobs you had performed in the health and social care sector?
- What motivated you to **carry on** being a HomeHealth worker?

**Service delivery**

- How would you **describe your experience** of delivering the Homehealth service?
- Can you talk me through the **approach** you typically took with participants?
  - Can you describe a typical **initial session** with a participant?
  - What about a **follow-up session**?
- Can you describe a **typical last session** with a participant?
- When ending the service for a participant, how did you **encourage maintenance** of behaviour?
  - - What **resources** were available to support clients with this? (e.g., access to local groups, carer support, written maintenance plan).
    - What were the **challenges** with this? How did you try and overcome them?
- How did you find the **length** of the appointments (FU visits expected to last 30-60mins ended up around 90mins)? How long were they? Why were they longer?
- What **worked well** in the delivery of HomeHealth? Can you give me an example?
  - How well did **participants engage** with the service?
- What **didn’t work** as well in the delivery of HomeHealth? Can you give me an example? (e.g., poor engagement)
  - Were there any **specific challenges to participants engagement** (e.g., working around holidays, hospitalisations)?
    - Did you find that **distinct groups/clusters** emerged?
  - What were the **challenges** you faced when delivering the HomeHealth service? (Prompts: Admin, goal attainment scaling, managing caseload, travel, participant engagement, participants not understanding things like goal setting)
  - What **challenges** did participants report to you? (i.e., identifying and setting goals, barriers when working towards their goals)
  - How did you **overcome** these challenges?
- Was there anything you would **do differently** with hindsight to overcome the challenges?
- How did you find the process of **identifying and setting goals** with participants? (Prompt: use of Health and wellbeing plan, easiness of the process, participants’ reactions, language of goals).
  - Did any different **groups emerge**?
  - What were the most **common types of goals** set? [probe on different domains]
  - Were there **differences** in goal setting **between the different domains** (mobility, social, nutrition, psychological)? [prompts: types of goals set, willingness to set goals, ability to identify what to change]
  - What did you do if a person **did not achieve** their goal?
  - What did you do if they **achieved their goal** very well?
    - How common was to set up **new goals versus increasing the difficulty** of goals?
  - Did you work with any clients on **memory related goals**?
    - [If yes]: How did this go? How could it be improved?
- Did you experience any **safeguarding issues**?
  - [If yes] How did you respond? Can you share a safeguarding issue you faced?
    - Who did you refer the client to?
- How did you share the actions with the client?
- How did these issues impact on your delivery of the HomeHealth service?
- How well did it work when you needed to **share a participant** with another HomeHealth worker?
  - What could make this process go more smoothly?
- On reflection, overall is there anything you would do differently to improve the delivery of HomeHealth?

**Adaptations**

- Did you **need to** **adapt** the service? In what way?
  - For any particular groups of people? For different cultures? In what way?
- Did you need to use an **interpreter**? How did this go?
- What **challenges** arose when participants had **memory difficulties**? (prompts: choosing goals, goal progression etc) How did you overcome them?
  - How did you adapt the service when someone had **cognitive impairment or dementia**? Were there any differences in the way you delivered the intervention? (Prompts: carer involvement, memory goals, adapting goals). Following your experiences, is there anything you would do differently to improve this?
  - How was participants with cognitive impairment or dementia´s **engagement** compared to their peers?
  - What was the **impact** of HH for people with memory issues? Was there any difference in terms of how **beneficial** was it for them compared to their peers?
  - What **further training/signposting/resources** would have been useful to support people with memory difficulties?
- Many of our participants had **multiple health conditions**. What challenges arose in engaging people with more health problems? (prompts: booking appointments, choosing goals, progressing on/achieving goals)
  - How did you **manage**/ overcome this? [ask for each]
  - How did you **adapt** the intervention to their conditions?
    - Could you talk us through an example?
  - **Which combinations** of health conditions that seemed to have a greater impact? (e.g., depression/anxiety with a physical health condition, physical health conditions causing pain/breathing/mobility problems, dementia combined with other problems).
    - Why was this?
    - What (if any) particular adaptions did you have to make for these combinations?
    - How did **pain** impact? How did **mental health** impact?
- If you came across a **problem** with a participant that affected delivery of the HomeHealth service, how did you solve it?
  - Can you give me an example?
- Learning from your experiences, what might you do/recommend improving this with future clients?

**Covid-19**

- How did **Covid-19 affect** service delivery? (Prompts: practicalities, PPE, engagement, goals set)
  - How did Covid-19 affect the way you **organised** your work and **delivered** the intervention? (i.e., Travelling distances, taking time out for your children/family, exposure to covid, etc).
  - Particularly at the start, some appointments were remote. How did these **compare to face-to-face appointments**? (Prompts: rapport, tech issues, engagement, goal progress, paperwork, and logistics)

**Impact**

- What **feedback** did participants give you on what they thought of the service and how it affected them?
- What **changed for participants** you worked with as a result of HomeHealth?
- What was the **impact** of being involved in HomeHealth **on yourself**? (e.g., new skills/ways of working/confidence, career development etc).

**Setting**

- How well did HomeHealth **fit** into [voluntary sector organisation (VSO)]?
- How were you **integrated** into their working structures? (Prompts: team based in, meetings attended, training, peer support)
- How well did this **work for you**?
- How well did the HomeHealth **paperwork** fit with the systems used in [VSO]?
- How did you **balance** the different demands of HomeHealth (e.g., seeing participants, admin, travel)?
- How did HomeHealth fit around your **other commitments?** (i.e., life, family)

**Training**

- How did you find the **training course**?
  - What went well?
  - What could we improve?
- How did it fit with [VSO]’s **induction**?
  - What went well?
  - What could be improved?
- How long did it take you to **feel confident** delivering HomeHealth?
  - Were there any particular areas that proved **difficult**?
    - [If yes] How could the training be improved to address these?
  - How did you go about **getting started** on HomeHealth?
- What **other resources or support** would have been helpful for you initially? (i.e., Buddying or shadowing a more experienced HomeHealth worker)
- What **skills did you need to develop** for HomeHealth?
  - Are there any additional **skills you would have liked** to have been developed for HomeHealth?
    - [If yes] what are these? How could we support developing these?
- How did the **way you delivered** HomeHealth **change** as you got more experienced?

**Supervision**

- How did you find **supervision with other** HomeHealth workers?
  - What do you feel about the level of contact with HomeHealth workers working at the other sites to share problems /solutions?
  - What kinds of **issues** did you discuss **in group** supervision?
  - What kinds of **issues** did you discuss **in one-to-one** supervision?
- How did the **group dynamics** change as HomeHealth workers came and left?
- How did you find **supervision within [VSO]**?
  - (If not great) How should have VSO supervision been?
- How often did you **access topic experts**?
  - What kind of issues did you access them for? What did you think of their support/feedback?
  - (If not often) What would have **helped** you to **access** topic experts **more**?
- Is there any **extra support** that would have been helpful? If so, what would that be?
  - What (if any) areas you would have liked more guidance on?

**Future implementation**

- We would like to implement HH more widely in future. What **advice** would you give us?
  - Is there anything you would **change if** rolling this out **nationwide**? If so, what would that be? (most/least valuable parts of HomeHealth?)
- What do you think the **best set up** (e.g., part time, full time, caseload) would be for HomeHealth in future?
- What **skills** do you think a HomeHealth worker needs? What **training** should they have?
  - What **advice** would you give to a new HomeHealth worker starting in this role?

Is there anything else you would like to tell us?

**Thank you for your time and help.**

| Instructions for interviewer |
| --- |
| Switch audio recorder off |
| Posting voucher – suitable address requested |

**Topic guide for interviews with other stakeholders (e.g., service managers)**

[VSO] refers to the voluntary sector organisation – Age Uk Bradford, Age Uk Camden, HILS

*Thank you for agreeing to take part in an interview. We are interested in your experiences of hosting and supervising the HomeHealth service, both good and bad, so we can understand how we can best implement the service in future if effective. We will keep your responses as confidential as possible. You will not be identified by name or organisation in any publications, but as there are only a small number of organisations of who hosted HomeHealth, we cannot guarantee complete anonymity. You can stop the interview at any time and you do not have to answer all questions. We would like to audio record the interview. Do you have any questions before we start?*

| Instructions for interviewer |
| --- |
| Check consent form is completed |
| **Switch the audio recorder on** |

**General**

- Talk us through **how HomeHealth worked** in [VSO]
- Can you talk me through what you understood the **purpose of HomeHealth** service to be?
- What do you feel the **valuable parts** of HomeHealth were?
  - What were the **less valuable** parts?
- **Why** were you/VSO **interested** in being involved in HomeHealth?
- How does it **fit with the values** of [VSO]?
- How does it **differ from other services** delivered by [VSO]?

**Setup**

- Talk us through **how you set up HomeHealth** at [VSO]
- What **worked well** when setting up the service?
- What were the **challenges**?
  - Was an extra layer of administration/office resources required?
  - What did you do to **overcome the challenges**? Is there anything you would do differently with hindsight?
- What **procedures and protocols** needed to be developed?
- How easy was it to **recruit** HomeHealth workers and supervisors? What avenues did you use? Which worked well/did not work?
- Should the HomeHealth worker role be a **full or part time role** if implemented? Why?
- How did you decide **where to locate** HomeHealth workers and supervisors within [VSO]?
- Are there ways HomeHealth could have been **better integrated** into [VSO’s] systems?
- How long did it take for HomeHealth to be **running smoothly**?
- Did how HomeHealth was delivered at [VSO] **change** across the course of the study? In what way?
- Following your experience, what would you **recommend to improve** the set up or delivery of HomeHealth?

**Training**

- How long do you think it takes for someone to **feel confident delivering** HomeHealth?
- [if attended] What did you think of the **training**?
  - What went well?
  - How could it be improved?
- How did the training **fit** with your induction programme at [VSO]?
- Is there any **further training** that would have been helpful?

**Supervision [if applicable]**

- What kind of things did you **discuss** in HomeHealth workers supervision? How did you find this?
- What **worked well** for HomeHealth workers?
- What were HomeHealth workers main **challenges**?
- Most clients had **multiple health conditions** that potentially affected how they chose or worked on their goals. How was this addressed within supervision? Is there anything you would do differently with experience?
- Many clients had **memory problems** potentially impacting on how they engaged with the service – how was this addressed within supervision? Is there anything you would do differently?
- What were the **challenges of supervision**? How did you overcome these? With experience, what would you do differently?
- Would this **supervision model** work if implemented more widely? How might it need to be changed?
- Would **remote or face to face supervision** work well in future? Why?
- How did managing HomeHealth workers fit with your **other commitments**? Did it add to/detract from these?

**Going forwards**

- What would be **needed** for HomeHealth **to continue**? – resources
- Would HomeHealth be a service you would be **interested in delivering** in the future? Why?
  - Would you be prepared to offer the intervention across different areas?
- How would you envision HomeHealth working if **implemented more widely**? What would you change?
  - How to get people into the service (e.g., Who could refer participants to this service other than GPs?)
- Linking with GP practices and other local networks
- Number of HomeHealth workers, team setup, caseload
- Training
- Local supervision
- Expert supervision
- Administration
- Buy in needed
- How feasible would it be for you to **preserve the core service specification** if it was implemented more widely (i.e., number of home visits, keeping an individual intervention for patients with mild frailty)?

Is there anything else you would like to say?

**Thank you for your time and help.**

| Instructions for interviewer |
| --- |
| Switch audio recorder off |
