# Supplementary material for: Understanding how older people with mild frailty engage with behaviour change to support their independence: a qualitative study
Source: BMJ Open. 2025 Jan 30;15(1):e086642. doi: 10.1136/bmjopen-2024-086642 (PMC11784424; doi:10.1136/bmjopen-2024-086642)
Supplement: online supplemental material 2 [file bmjopen-15-1-s002.docx]

# Supplementary material C – Further details on study team and reflexivity

The study team consisted of a range of disciplines/backgrounds (psychology, public health, health services research, general practice, nursing, and public contributors with lived experience), each bringing unique insights to the interpretations of findings. The study team included three males (BG, PC, RK) aged between 26-70 years old. Most members had an interest in improving health and well-being in older people. Several were independent to the main trial, including the process evaluation lead (YBM) and team members undertaking and coding the interviews. Researchers conducting interviews completed a research diary after each interview to record their observations and impressions.

Researchers conducting the interviews all were all at post-graduate or post-doctoral level, their highest qualifications ranged from MSc to PhD, and had between 1-10 years’ experience conducting interviews. Interviews with HomeHealth workers and key stakeholders were conducted by one researcher (YBM). No interviews were repeated.

Researchers conducting interviews did not know older participants before the interviews, however, time was allocated before interviews to develop some rapport with the participant. Researchers introduced themselves prior to the interview, explained their role in the study, the rationale to do the interviews and highlighted there were no right or wrong answers to facilitate an open conversation.
