# Supplementary material for: Understanding how older people with mild frailty engage with behaviour change to support their independence: a qualitative study
Source: BMJ Open. 2025 Jan 30;15(1):e086642. doi: 10.1136/bmjopen-2024-086642 (PMC11784424; doi:10.1136/bmjopen-2024-086642)
Supplement: Supplementary file 4 [file bmjopen-2024-086642.reviewer_comments.pdf]

PEER REVIEW HISTORY

BMJ Open publishes all reviews undertaken for accepted manuscripts. Reviewers are asked to complete a checklist review form and are provided with free text boxes to elaborate on their assessment. These free text comments are reproduced below.

ARTICLE DETAILS

Title (Provisional)

Understanding how older people with mild frailty engage with behaviour change to support their independence: A qualitative study

Authors

Barrado-Martín, Yolanda; Frost, Rachael; Catchpole, Jessica; Rookes, Tasmin Alanna; Gibson, Sarah; Avgerinou, Christina; Gardner, Benjamin; Gould, Rebecca L.; Chadwick, Paul; Hopkins, Jane; Drennan, Vari M.; Kharicha, Kalpa; Marston, Louise; Kumar, Rashmi; Elaswarapu, Rekha; Jowett, Claire; Walters, Kate R

VERSION 1 - REVIEW

**Reviewer**	**1**
**Name**	**Finnegan, Susanne**
**Affiliation**	**University of Warwick**
**Date**	**03-May-2024**
**COI**	**No competing interests**

Thank you for submitting this qualitative work to understand how older people with mild frailty engage with behaviour change to support their independence.

Page 5 - Strengths and limitations

Line 25: I think this is a typo - ...and differentiate or service from other services received.

Introduction: clear rationale and background for the study.

Methods:

Page 7, line 42 - Montral Cognitive Assessment - is this a typo - should be Montreal?

Page 8 - 9, lines 35 onwards - a lot of what you are describing in the 'interviews' section are actually results rather than methods and so this should be moved to the results section. You will need to amend this section to just describe what you did during the interviews i.e., how participants gave consent and whether interviews were over the phone or Teams or F2F etc.

Page 9 - the last two sentences of the 'analysis' section also feel like results rather than methods and need to be moved accordingly.

Results:

Theme 1: Really interesting to hear that some struggled to make goals and were already active with hobbies or socialising - can you elaborate as to why or how they came to be involved in the trial if this was the case - what was the eligibility criteria for the trial?

Discussion:

Page 18, lines 31 to 34 - this paragraph feels a bit limited and needs more detailed as to where these examples of issues with goal setting came from and how they compare to your findings.

Reviewer 2

Name Silies, Katharina

Affiliation University of Lübeck, Institute for Social Medicine and Epidemiology, Nursing Research Unit

Date 09-Jul-2024

COI I declare no competing interests

Dear authors, I have been very pleased to have the opportunity to review your manuscript.

You present results of a qualitative evaluation strand in an intervention study based on methods designed for the process evaluation of this study. Your report is well written, and adheres mostly to the relevant reporting guidelines. You present four overarching themes on the engagement of older and frail persons with a behavior change approach to support independent living, following the aim of your paper to explore what influences engagement with behavior change approaches in older people with mild frailty and how this can be facilitated.

My main concern is the separate reporting of results partly in this paper (4 themes) and partly in the process evaluation paper (2 themes). It is important to justify why you would want to separately report results of a single analysis of a single data base in two papers. Currently, themes presented here partly overlap with aspects I would expect in a process evaluation paper, as they offer explanations for mechanisms of change. The description of the intervention is provided in a very short manner in the background, which makes it difficult to understand some of the results and conclusions you present. I understand the additional value of an in-depth reflection of results that emerge during an effectiveness trial where it is not always possible to present everything in detail. But the rationale must be well argued for and reported transparently. In principle, I can support your approach if the results in this paper can be generalized sufficiently (meaning your data allows for this and limitations are discussed). To place a stronger focus on the COM-B model might be a way to do this. Meaning: I recommend to apply the dimensions of the COM-B as an overarching structure for your themes, explaining how each of the dimensions were addressed by the

intervention, summarizing results to the domain and interpreting and discussing the results with regard to the applicability of the model.

Thus, you could give specific insights of how to handle the model in complex interventions addressing behavior change which would be of interest to the scientific community as well as to intervention providers in practice.

Detailed review:

Major comments (content)

Abstract:

1. L21: … a behavior change intervention – I suggest to add some words on what kind of intervention this is.

2. L24: minor comment: I recommend to number the themes. … were identified: 1. Engagement with …; 2. Goal progress …

3. L43: Regular and continued … - It is unclear, what the intervention looks like, therefore I find it a bit difficult to understand that this may have been a key component.

4. L45: … wanting to change their behavior. – I recommend to express the target behavior.

Strengths and Limitations:

5. L25 … differentiate our service from other … - typo?

Introduction/Background:

6. L22: We co-designed a health promotion service … - I think, more information on this is needed, e.g. the intervention components and how the service is implemented. You present a very nice TIDieR template with your study protocol, I recommend to mention this here explicitly. A short table on your intervention would still be appreciated highly by readers, I could imagine.

7. Aim: … what influences engagement with …. This aim is very close to the research question of a process evaluation. Your aim should express clearly, what you want to achieve by this additional paper and why it is justified that you may reach this aim by working with results from a process evaluation study.

Methods:

8. L43: MS Teams: please add reference for the software.

Results

9. Please provide a code book containing your coding tree, a short description / definition of each code and an anchor quotation. This can be added as a supplement. The code book should contain all 6 themes from your analysis.

10. L10: We identified four overarching themes … - This should be specified, as you identified 6 themes but only report 4 here. A short table with an overview of the themes and subthemes would also be helpful to the reader.

11. Currently, aspects in themes are seperated, that I would expect to be summarised with reference to the elements of the COM-B Model. What kind of activities did the intervention include to address each of the domain? How did participants respond to that? What was supportive, what was hindering? How should this be interpreted for future interventions based on the COM-B model? As stated above, I therefore recommend to reorganize your results according to the dimensions of the COM-B model.

Discussion:

12. I recommend to follow the dimensions of the COM-B model also in the introductory summary of your results which opens the discussion part. Currently, the discussion is a bit fragmented, picking up isolated (partly) results but lacking a visible read thread.

VERSION 1 - AUTHOR RESPONSE

Reviewer: 1
Dr. Susanne Finnegan, University of Warwick
Comments to the Author:
Thank you for submitting this qualitative work to understand how older people with mild frailty engage with behaviour change to support their independence.

Page 5 - Strengths and limitations
Line 25: I think this is a typo - ...and differentiate or service from other services received.

Thank you, we have now corrected this.

Introduction: clear rationale and background for the study.

Thank you.

Methods:
Page 7, line 42 - Montral Cognitive Assessment - is this a typo - should be Montreal?

We have now corrected this.

Page 8 - 9, lines 35 onwards - a lot of what you are describing in the 'interviews' section are actually results rather than methods and so this should be moved to the results section. You will need to amend this section to just describe what you did during the interviews i.e., how participants gave consent and whether interviews were over the phone or Teams or F2F etc.

We have moved the sections on recruitment and numbers of participants to the results, 'participant characteristics' section.

Page 9 - the last two sentences of the 'analysis' section also feel like results rather than methods and need to be moved accordingly.

We have deleted these.

Results:
Theme 1: Really interesting to hear that some struggled to make goals and were already active with hobbies or socialising - can you elaborate as to why or how they came to be involved in the trial if this was the case - what was the eligibility criteria for the trial?

We have amended the results substantially in light of reviewer 2's comments, so this data is now in 'Motivation', Perceived need for Change theme. Older people aged 65+ with mild frailty, who were not already case managed or receiving palliative care, were invited to participate. We did not screen for having goals or readiness to change, and so some participants reported joining the trial for altruistic reasons, to help research, rather than from a defined need.

Discussion:
Page 18, lines 31 to 34 - this paragraph feels a bit limited and needs more detailed as to where these examples of issues with goal setting came from and how they compare to your findings.

We have now expanded this section with more information on the two cited studies and how they relate to our findings.

Reviewer: 2
Dr. Katharina Silies, University of Lübeck
Comments to the Author:
Dear authors, I have been very pleased to have the opportunity to review your manuscript.
You present results of a qualitative evaluation strand in an intervention study based on methods designed for the process evaluation of this study. Your report is well written, and adheres mostly to the relevant reporting guidelines. You present four overarching themes on the engagement of older and frail persons with a behavior change approach to support independent living, following the aim of your paper to explore what influences engagement with behavior change approaches in older people with mild frailty and how this can be facilitated.
My main concern is the separate reporting of results partly in this paper (4 themes) and partly in the process evaluation paper (2 themes). It is important to justify why you would want to separately report results of a single analysis of a single data base in two papers. Currently, themes presented here partly overlap with aspects I would expect in a process evaluation paper, as they offer explanations for mechanisms of change. The description of the intervention is provided in a very short manner in the background, which makes it difficult to understand some of the results and conclusions you present. I understand the additional value of an in-depth reflection of results that emerge during an effectiveness trial where it is not always possible to present everything in detail. But the rationale must be well argued for and reported transparently. In principle, I can support your approach if the results in this paper can be generalized sufficiently (meaning your data allows for this and limitations are discussed). To place a stronger focus on the COM-B model might be a way to do this. Meaning: I recommend to apply the dimensions of the COM-B as an overarching structure for your themes, explaining how each of the dimensions were addressed by the intervention, summarizing results to the domain and interpreting and discussing the results with regard to the

applicability of the model.
Thus, you could give specific insights of how to handle the model in complex interventions addressing behavior change which would be of interest to the scientific community as well as to intervention providers in practice.

Thank you for this comment. The sister process evaluation paper reports on a mixed-methods process evaluation specific to the intervention and trial, incorporating quantitative data on population reach, fidelity, goals set and quantitative exploration of mechanisms of impact, in addition to some qualitative data on acceptability of the intervention and contextual factors affecting this. Given the wealth of information in the sister paper, we were very restricted in what we could report from the substantive qualitative study we conducted, which had generated rich data on behaviour change in this population more generally. We had further explored the processes of behaviour change and factors affecting this in a way that is more transferable to other similar interventions, and themes generated from this data is what is reported in this current paper. We appreciate the reviewer's suggestion to structure by COM-B. We have reorganised the results to follow this structure, and have added further detail on how HomeHealth workers attempted to overcome barriers and the extent to which these were successful. Please note we have not used tracked changes for this section (the results new themes section) due to the large volume of changes made in order to restructure the themes using COM-B as advised.

Detailed review:
Major comments (content)
Abstract:
1. L21: … a behavior change intervention – I suggest to add some words on what kind of intervention this is.

We have now added further details. Please note this was limited by the word count.

2. L24: minor comment: I recommend to number the themes. … were identified: 1. Engagement with …; 2. Goal progress …

The themes have been changed in light of your feedback regarding COM-B. As such, we have not numbered the themes but hope they are clearly represented under their COM-B headings.

3. L43: Regular and continued … - It is unclear, what the intervention looks like, therefore I find it a bit difficult to understand that this may have been a key component.

We hope that the clarification in point 1 above has added further context for this statement.

4. L45: … wanting to change their behavior. – I recommend to express the target behavior.

This was a personalised intervention and therefore the target behaviour differed from person to person. As such, we cannot specify this.

Strengths and Limitations:
5. L25 … differentiate our service from other … - typo?

Thank you, we have amended this.

Introduction/Background:
6. L22: We co-designed a health promotion service … - I think, more information on this is needed, e.g. the intervention components and how the service is implemented. You present a very nice TIDieR template with your study protocol, I recommend to mention this here explicitly. A short table on your intervention would still be appreciated highly by readers, I could imagine.

We have reorganised this section so the components of the service are clearer and have more explicitly referenced where the reader can find more information, in both our HTA report (in detail) and in our protocol paper/TIDieR checklist. We have not currently included an additional table of the intervention content within this paper, as we are aiming to consider the barriers and facilitators to behaviour change in this population more generally and how barriers can be addressed, and not focus specifically on the intervention itself in detail.

7. Aim: … what influences engagement with …. This aim is very close to the research question of a process evaluation. Your aim should express clearly, what you want to achieve by this additional paper and why it is justified that you may reach this aim by working with results from a process evaluation study.

We have clarified that this paper aims to outline barriers and facilitators to behaviour change and the how successful strategies were to overcome these. We have also changed the abstract aim to reflect this.

Methods:
8. L43: MS Teams: please add reference for the software.

We have changed MS Teams to state video-conferencing instead for clarity.

Results
9. Please provide a code book containing your coding tree, a short description / definition of each code and an anchor quotation. This can be added as a supplement. The code book should contain all 6 themes from your analysis.

We followed Braun & Clarke's (2021) description of codebook thematic analysis, which uses a structured coding framework for developing and documenting analysis, but that can be refined and new themes developed through inductively engaging with the data and analytic process. As such, we developed an initial set of codes across the whole dataset. This was generated through inductive coding of a sample of transcripts, review by the qualitative process evaluation team, and applying to the wider dataset. Analyses then used different combinations of codes to answer different research questions. As our codebook was the first step in an iterative analysis process we are uncertain if this would be informative for the readers. It is very long (100 pages) and comprehensive with detailed initial coding, not all of which is relevant to the themes of this paper. We have not had to provide this for qualitative papers before using this approach.

Virginia Braun & Victoria Clarke (2021) One size fits all? What counts as quality practice in (reflexive) thematic analysis?, Qualitative Research in Psychology, 18:3, 328-352, DOI: 10.1080/14780887.2020.1769238

10. L10: We identified four overarching themes … - This should be specified, as you identified 6 themes but only report 4 here. A short table with an overview of the themes and subthemes would also be helpful to the reader.

In response to the earlier comment we have now substantially restructured the themes around COM-B headings. We have included our inductive themes structured under each of the COM-B headings, which required some reorganisation of the content. We have not currently included a summary table in addition, as addressing the comments has increased the length of the paper but can include this if the editors feel this is required.

11. Currently, aspects in themes are seperated, that I would expect to be summarised with reference to the elements of the COM-B Model. What kind of activities did the intervention include to address each of the domain? How did participants respond to that? What was supportive, what was hindering? How should this be interpreted for future interventions based on the COM-B model? As stated above, I therefore recommend to reorganize your results according to the dimensions of the COM-B model.

We have reorganised our themes accordingly to outline what hindered and facilitated behaviour changes according to the COM-B structure, and have expanded on ways the HomeHealth workers tried to overcome these, and the extent to which they were successful.

Discussion:
12. I recommend to follow the dimensions of the COM-B model also in the introductory summary of your results which opens the discussion part. Currently, the discussion is a bit fragmented, picking up isolated (partly) results but lacking a visible read thread.

We have revised the opening section of the results to follow the dimensions of the COM-B model and is more directly aligned to the re-structured findings section.

Reviewer: 1
Competing interests of Reviewer: No competing interests

Reviewer: 2
Competing interests of Reviewer: I declare no competing interests

Other amendments:

We identified that the manuscript was missing the reference 21 Braun and Clarke 2021 from the reference list, which has now been added.

VERSION 2 - REVIEW

**Reviewer**	**1**
**Name**	**Finnegan, Susanne**
**Affiliation**	**University of Warwick**
**Date**	**30-Oct-2024**
**COI**	

Thank you very much for addressing all of my comments and I appreciate the substantial changes you have made.

**Reviewer**	**2**
**Name**	**Silies, Katharina**
**Affiliation**	**University of Lübeck, Institute for Social Medicine and Epidemiology, Nursing Research Unit**
**Date**	**17-Nov-2024**
**COI**	

Dear authors, I have been very pleased to have the opportunity to review your revised manuscript.

You have made substantial revisions and addressed all reviewers' comments comprehensively and adequately. I am very pleased that you found it feasible to arrange your results according to the COM-B model and appreciate the now clear structure of your results section.

I have no further concerns.

Minor comments:

P10: …they were physical unable to achieve a goal. – Please check grammar (physically?)

Quotes: I recommend to start quotes with a capital letter.

Good luck with your manuscript!
